# Study protocol: effects of school gardens on children’s physical activity

**DOI:** 10.1186/2049-3258-72-43

**Published:** 2014-12-08

**Authors:** Nancy M Wells, Beth M Myers, Charles R Henderson

**Affiliations:** Design & Environmental Analysis Department, College of Human Ecology, Cornell University, Ithaca, NY 14853 USA; Department of Human Development, College of Human Ecology, Cornell University, Ithaca, NY 14853 USA

**Keywords:** Children, Gardens, Physical activity, Sedentary behavior, Health behaviors, Schools, Randomized controlled trial

## Abstract

**Background:**

Childhood obesity is an epidemic. Strategies are needed to promote children’s healthy habits related to diet and physical activity. School gardens have the potential to bolster children’s physical activity and reduce time spent in sedentary activity; however little research has examined the effect of gardens on children’s physical activity. This randomized controlled trial (RCT) examines the effect of school gardens on children’s overall physical activity and sedentary behavior; and on children’s physical activity during the school day. In addition, physical activity levels and postures are compared using direct observation, outdoors, in the garden and indoors, in the classroom.

**Methods/Design:**

Twelve New York State schools are randomly assigned to receive the school garden intervention or to serve in the wait-list control group that receives gardens and lessons at the end of the study. The intervention consists of a raised bed garden; access to a curriculum focused on nutrition, horticulture, and plant science and including activities and snack suggestions; resources for the school including information about food safety in the garden and related topics; a garden implementation guide provided guidance regarding planning, planting and maintaining the garden throughout the year; gardening during the summer; engaging volunteers; building community capacity, and sustaining the program.

Data are collected at baseline and 3 post-intervention follow-up waves at 6, 12, and 18 months. Physical activity (PA) “usually” and “yesterday” is measured using surveys at each wave. In addition, at-school PA is measured using accelerometry for 3 days at each wave. Direct observation (PARAGON) is used to compare PA during an indoor classroom lesson versus outdoor, garden-based lesson.

**Discussion:**

Results of this study will provide insight regarding the potential for school gardens to increase children’s physical activity and decrease sedentary behaviors.

**Trial registration:**

Clinicaltrial.gov # NCT02148315

**Electronic supplementary material:**

The online version of this article (doi:10.1186/2049-3258-72-43) contains supplementary material, which is available to authorized users.

## Background

In the context of the epidemics of both childhood obesity [1] and children’s physical inactivity [2–4], strategies are needed to improve children’s health behaviors. Physical activity (PA) has been linked to lower likelihood of chronic diseases such as overweight and obesity, cardiovascular disease, osteoporosis, and type 2 diabetes [5–8]. Conversely, a lifestyle of sedentary behaviors is associated with the onset of chronic diseases, disabilities, and early-life mortality [9, 10]. Fruit and vegetable consumption is similarly linked to reduced disease risk [11–14]. Schools are increasingly recognized as a promising context for health intervention [15, 16]. A variety of school-based interventions have aimed to increase PA through, for example, increased physical education time, added recess periods, painted playground surfaces, strategies to reduce TV and computer game usage, and dance programs [17, 18]; however little research has focused on the possible role of school gardens in the promotion of children’s health behaviors. School gardens have the potential to affect both PA behaviors and dietary intake as well as to contribute to learning outcomes [19, 20].

School gardens typically include an instructional garden plot and accompanying garden-based lessons. Gardens have been used to teach a variety of subjects including science, environmental studies, nutrition, language arts, and math [20]. While we know that time outdoors is a strong predictor of physical activity [21, 22], there is need for research explicitly examining the effects of school gardens on children’s PA, using valid, objective measures of PA. Gardens may increase PA by bringing typically indoor, sedentary activities outdoors and by engaging children in hands-on, garden-based learning tasks.

## Research questions

This project addresses the following specific research questions (adjusting for relevant sociodemographic variables):

1. Is there an effect of school gardens on children’s overall PA and sedentary behavior, as measured by self-report survey?
2. Is there an effect of school gardens on children’s PA levels during the school day, as measured with accelerometry?
3. In a within-subjects comparison, does PA, measured by direct observation, differ during an indoor classroom lesson versus during an outdoor garden lesson?

## Methods/Design

### Study design

**Figure 1:**
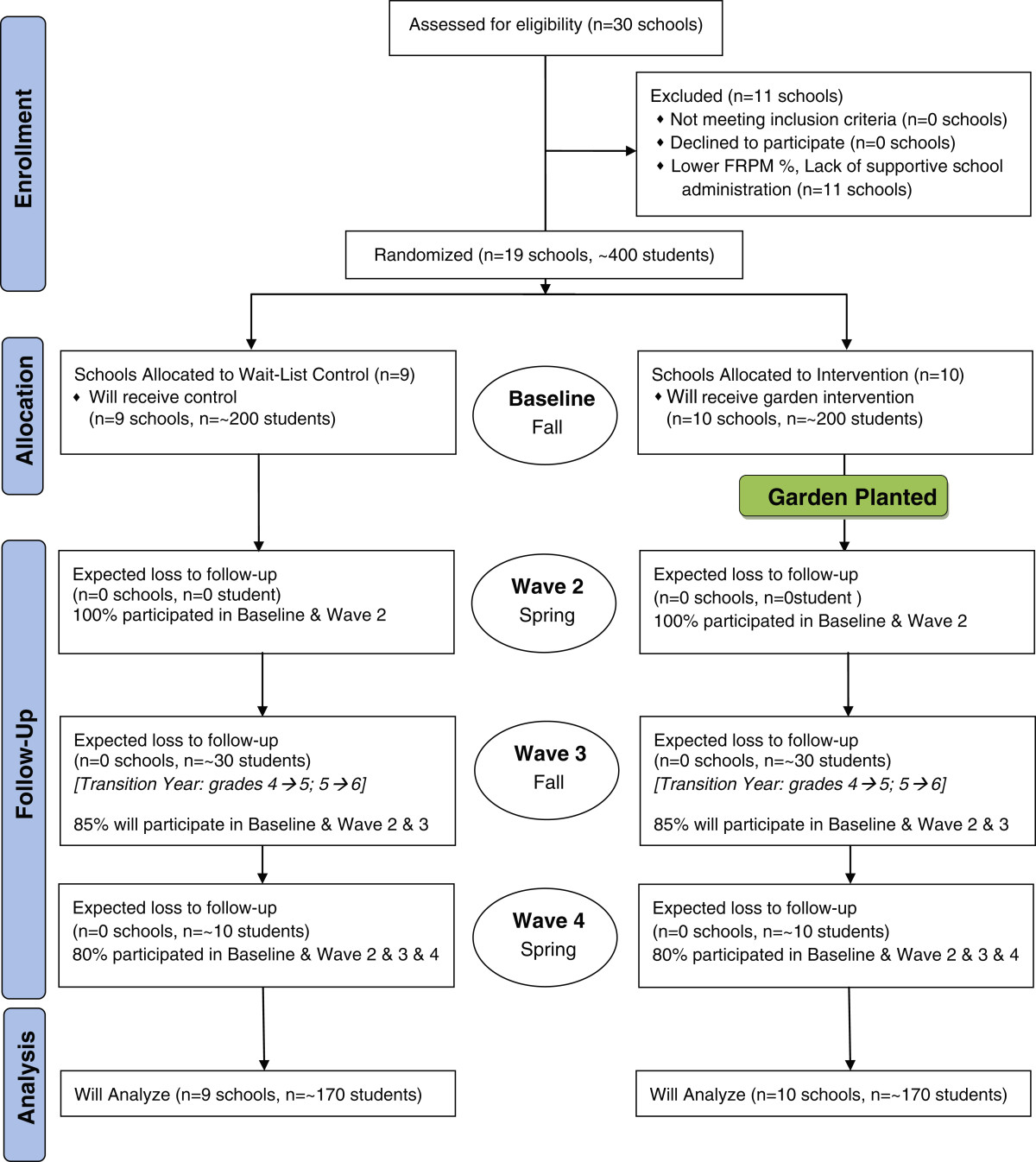
**Flow diagram for school gardens physical activity RCT.**

The study is a 2-year, randomized controlled trial, with baseline and three follow-ups at ~6 months, ~12 months, and ~18 months (see Figure 1). Schools are randomly assigned either to receive the garden intervention or to serve in the wait-list control group that receives the garden and garden-based curriculum at the end of the study. For both intervention and control schools, the receipt of a school garden is an incentive for participation. The study is registered with ClinicalTrials.gov, #NCT02148315. The research design and methods were deemed exempt by Cornell University’s Institutional Review Board (IRB).

### Intervention

This study piggybacks on the larger, *Healthy Gardens, Healthy Youth (HGHY)* 4-state study of school gardens’ effects on diet and related outcomes that, through USDA funding, provides the garden and curricula intervention. Classes in the garden intervention group receive: a) a 4′ × 8′ raised bed garden kit; b) an educational toolkit containing lessons focused on nutrition, horticulture, and plant science (11 lessons for grades 4–5 in Year 1; and 9 lessons for grades 5–6 in Year 2), activities (e.g., songs, tastings, observations, journaling, role playing), and suggested recipes; c) a garden implementation guide that provides instruction on topics such as: how to plan, plant, and maintain the garden; gardening during the summer; engaging volunteers; building community capacity; and sustaining a school garden program; and d) on-line video training and documentation on how to use the toolkit. Lessons are led either by the classroom teacher or a Cooperative Extension Educator. Classrooms in the control group receive a garden and access to the educational toolkit after completion of data collection.

### Setting, sample size, design and randomization

School principals were approached by local Cooperative Extension educators. Data are collected in New York State elementary schools that do not already have garden and have, at least, 50% of students qualifying for free or reduced price meals (FRPM). During the study enrollment process, 30 New York State schools were screened for eligibility. Of the 30 schools, 19 met the inclusion criteria and were randomly assigned to intervention (n = 10) or control (n = 9). Data are collected at four time points (waves) during the project: a baseline assessment, and at three follow-ups. During each wave, we track the expected loss of schools and students (Figure 1) across the study. Fourth and fifth grade students (ages 8–12 at baseline) participated in this study.

### Compliance with CONSORT

CONSORT guidelines [23, 24] will be followed for presentation of results from randomized controlled trials. We will summarize the flow of both schools and individuals through the trial.

### Data collection methods

Children’s PA is measure in three ways: 1) the Girls Health Enrichment Multi-site (GEMS) Activity Questionnaire (GAQ); 2) Accelerometry; and 3) Direct Observation; with each measure corresponding to one of the research questions.

#### Activity questionnaire

The GAQ [25] is administered in the classroom to measure children’s overall physical activity and sedentary behavior. The GAQ, which is derived from the Self-Administered Physical Activity Checklist (SAPAC), has been validated with heart rate (r = .57, p < .001) and accelerometry (r = .30, p < .001) with 125 5^th^ graders [26]. The GAQ gathers information about children’s PA and sedentary activity “yesterday” and “usually” in 28 sports and PAs (e.g., bicycling, volleyball, gymnastics) and 7 sedentary activities (e.g., watching TV or videos; playing computer games; playing board games; listening to music).

#### Accelerometry

Children wear Actigraph GT3X + or GT1M accelerometers during the entire (~6 hour) school day. In use with children, accelerometry data are highly correlated with energy expenditure (r = .86, .87), oxygen consumption (r = .86, .87), heart rate (r = .77, .77), and treadmill speed (r = .90, .89) [27, 28]. In the morning, trained Cooperative Extension educators or University student research assistants distribute the accelerometers to the children and record the belt numbers and time of day. With assistance, children attach the accelerometers to their waists with an elastic belt and plastic buckle and are instructed to follow their normal routine. Teachers are given instructions on how to ensure children properly wear the accelerometers for the entire school day. At the end of each school day, classroom teachers collect the accelerometers and record the time of day. This procedure is followed for three days at each of the four waves of data collection.

#### Direct observation

To characterize children’s movements and postures during a garden lesson compared to a classroom lesson, the Physical Activity Research & Assessment tool for Garden Observation (PARAGON) [29] is employed. PARAGON’s overall test-retest reliability is .94. An Ebel of .97 (and percent agreement 88%) indicates strong inter-rater reliability [29]. The five primary PA codes (lying, sitting, standing, walking, vigorous activity) used in PARAGON are based on Behaviors of Eating and Activity for Children’s Health (BEACHES) PA coding and were validated using heart-rate monitors and accelerometers [30, 31]; and by convergent validity with accelerometry [29].

### Process measures

To record the fidelity of the intervention delivered to each intervention class, a “garden records” survey is completed by the Extension Educator. Garden records are completed at each intervention wave and include: fruits and vegetables (FV) planted, FV harvested, the methods employed to distribute FV, and the lessons delivered to the class.

### Statistical models and data analysis strategy

Treatment (control versus intervention) and time of assessment (Waves 1–4) are core fixed classification factors. The sampling design results in a nested structure. Schools are nested within fixed factors built into the sampling design—urban/rural status; classrooms are nested within schools, and children within classrooms. These 3 classification factors are regarded as random. Other classification factors and covariates that will be included in models, or at least examined for inclusion, include sex, race/ethnicity, and age of child, and school-level, and classroom-level characteristics. An important focus of analysis will be on the contextual effects of classrooms and schools and the moderation of the effects of the intervention by these contexts. Time in PE and recess are key classroom-level covariates.

The 3 research questions will be examined in models of the preceding type. The examination of treatment effects focuses on the treatment–time interaction. We will also examine in detail whether treatment effects are stronger for or limited to certain child or contextual characteristics. Important sociodemographic variables such as sex and ethnicity of the child will be included in the models irrespective of their moderating effects and the treatment differences adjusted for these variables.

The primary analyses will make use of general linear mixed model methods and their extensions to examine the research questions. The focus will be on (1) full model specification to account for all sources of variation, and a full examination of interactions among model factors, including examination of homogeneity of regressions to understand interactions between classification effects and covariates; (2) mixed models to take into account variances associated with children and families as well as classrooms, schools, and communities, to analyze random regressions associated with children and other levels of the analysis, and to examine contextual effects of classrooms and schools; (3) mixed models (including repeated measures and growth curves) to analyze multiple assessments on children; (4) generalized models to analyze dichotomous outcomes with binomial error distributions and count data assumed to have Poisson or negative binomial distributions; and (5) semi-parametric methods to model relations between variables without requiring a specific functional form. Generalized models will be analyzed in mixed model form when random factors are included.

Accelerometry data are scored using ActiLife6 software. Thirty-second epochs [32] are converted into minutes and proportions of time spent in each of the four levels of PA: 1) sedentary, 2) light PA, 3) moderate PA, 4) vigorous PA; and moderate-to-vigorous PA (MVPA) using child-specific cut-points [33, 34].

### Statistical power

Power calculations carried out for the original grant application indicate sufficient sample size to detect meaningful treatment mean differences at the level of a 2-way interaction for outcomes.

## Discussion

The limitations of this study include a focus on New York State youth, which limits generalizability. In addition, the garden intervention is examined holistically, so the specific activities that may be linked to increases in PA levels are not identified.

Results of this RCT will fill a gap in research literature and may provide insight regarding the potential for school gardens to increase children’s physical activity and decrease sedentary behaviors. By employing random assignment, using multiple measures, and providing longitudinal data over a 2-year period, this study will be the first to assess rigorously causal links between school gardens and children’s physical activity.

## Authors’ original submitted files for images

Below are the links to the authors’ original submitted files for images. Authors’ original file for figure 1

## Abbreviations

RCT: Randomized controlled trial
PA: Physical activity
FRPM: Free and reduced price meals
HGHY: Healthy gardens healthy youth
GEMS: The Girls Health enrichment multi-site study
GAQ: GEMS activity questionnaire
SAPAC: Self-administered physical activity checklist
PARAGON: Physical activity research & assessment tool for garden observation
FV: Fruit and vegetables
MVPA: Moderate-to-vigorous physical activity.
