# Supplementary figures and images for: Study protocol: effects of school gardens on children’s physical activity
Source: Arch Public Health. 2014 Dec 8;72:43. doi: 10.1186/2049-3258-72-43 (PMC4322466; doi:10.1186/2049-3258-72-43)

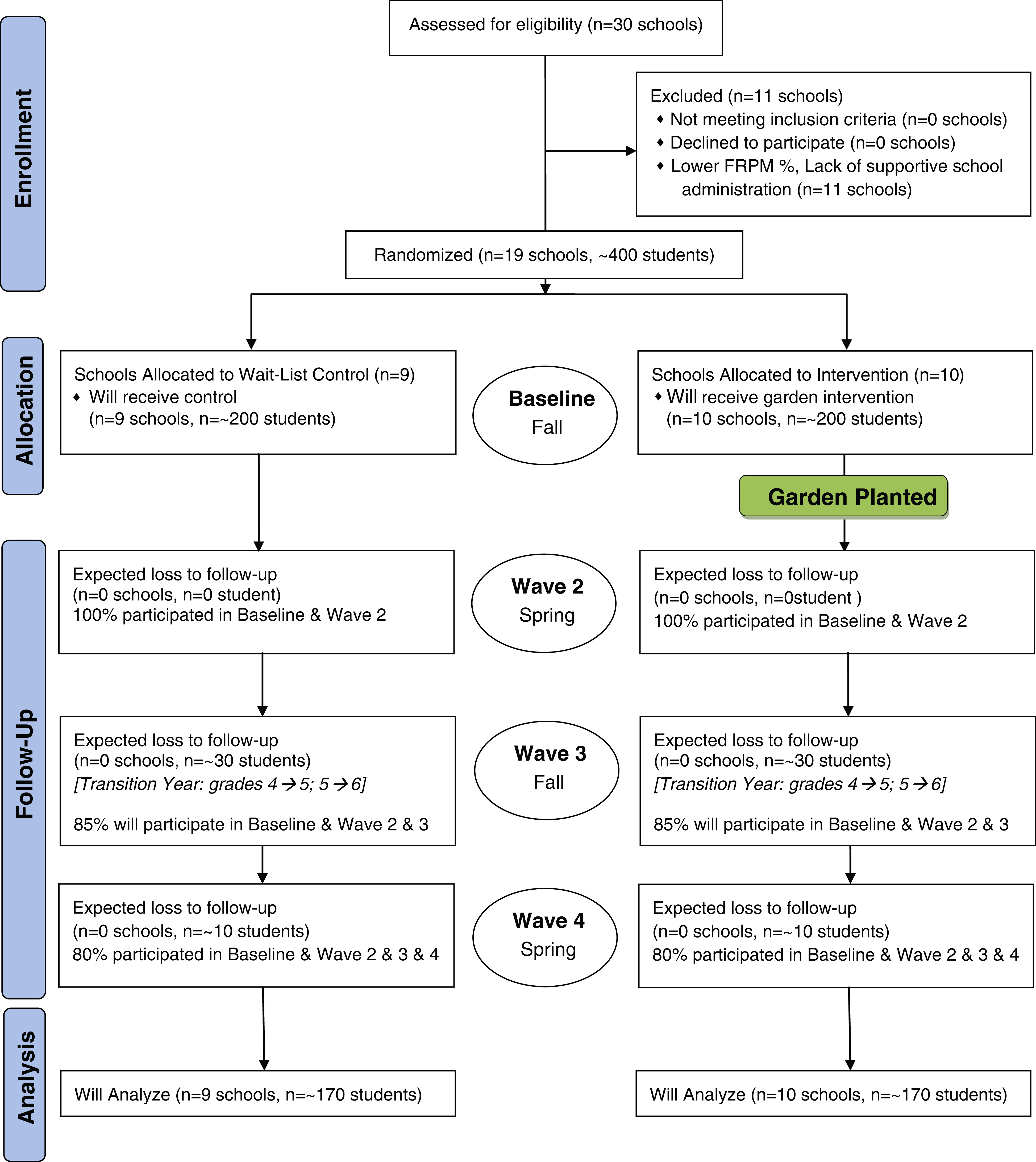

Supplement: Supplementary file 1 — Authors’ original file for figure 1 [file 13690_2014_5056_MOESM1_ESM.tif]
